# Parliament and the Professions

**Published:** 1920-05-22

**Authors:** 


### Parliament and the Professions.

### The Financing of the Voluntary Hospitals.

IN the House of Commons on Wednesday, May 19, Sir C. Kinloch Cooke asked the Minister of Health whether he is now in a position to make some definite statement regarding the steps he is taking to meet the difficulty that has arisen in connection with the finance of London hospitals; whether he is aware that some hospitals are even now unable to meet their current liabilities; and that unless relief by grants in aid is immediate they will have no alternative but to curtail their munificent work, and possibly close down altogether.

Dr. Addison replied that he was in consultation with the various authorities, but could make no definite statement at present.

### Venereal Diseases in the Army.

MR. CHURCHILL stated that there had been a rise in the incidence of venereal disease amongst the troops in France and Germany since the Armistice. The subject has received anxious consideration for some months past, and he was satisfied that the authorities on the spot are taking all possible steps to combat it. In answer to another question, Mr. Churchill said that no orders have been given by the military authorities recognising houses of ill-fame.

### Indian Medical Service.

MR. MONTAGU stated that in 1914 the number of officers in the Indian Medical Service was 706 Europeans and 63 Indians. In December 1919, excluding officers holding temporary commissions, there were 650 Europeans and 80 Indians. During 1919, 25 Europeans and 21 Indians were appointed to permanent commissions. He hoped soon to announce increased rates of pay and pension, and he recognised that it is essential to improve the facilities for leave and study, but no decision on these points can be effective until recruitment has brought the Service nearer to its normal strength.

### Charitable Donations and Income Tax.

MR. WATERSON asked the Chancellor of the Exchequer whether the United States Government have made special provision to meet the needs of charitable institutions, so as not to allow such donations to be commuted for income-tax purposes; and, if such is done, is he prepared to consider such a policy for this country. Mr. Chamberlain replied that the matter was considered by the Royal Commission on the Income Tax, but they were unable to recommend that such an allowance should be made in the United Kingdom. The United States income-tax law allows a deduction from the taxable income of an individual of contributions or gifts to corporations organised and operated exclusively for religious, charitable, scientific, or educational purposes, or for the prevention of cruelty to children or animals, to an amount not exceeding 15 per cent. of the taxpayer's net income as computed without the benefit of this provision. No deduction is allowed in computing the taxable income of a company or corporation.

